# Inland hypersaline lakes and the brine shrimp *Artemia *as simple models for biodiversity analysis at the population level

**DOI:** 10.1186/1746-1448-2-14

**Published:** 2006-11-28

**Authors:** Gonzalo M Gajardo, Patrick Sorgeloos, John A Beardmore

**Affiliations:** 1 Laboratory of Genetics & Aquaculture, Universidad de Los Lagos, P. O. Box 933, Osorno, Chile; 2 Laboratory of Aquaculture & Artemia Reference Center, Gent University, Rozier 43, Gent, Belgium; 3 John A. Beardmore, School of Medicine, University of Wales Swansea, Swansea SA2 8PP, UK

## Abstract

Biodiversity can be measured at different hierarchical levels, from genetic diversity within species to diversity of ecosystems, though policy-makers tend to use species richness. The 2010 goal of reducing biodiversity loss, agreed by the subscribers to the Convention on Biological Diversity, requires simple and reliable protocols to evaluate biodiversity at any level in a given ecosystem. Stakeholders, particularly policy makers, need to understand how ecosystem components interact to produce social and economic benefits on the long run, whilst scientists are expected to fulfil this demand by testing and modelling ideally simple (low diversity) ecosystems, and by monitoring key species. This work emphasizes the unique opportunity offered by inland, isolated salt lakes and the brine shrimp *Artemia*, an example of biodiversity contained at the intra-specific level, as simple models to understand and monitor biodiversity, as well as to assess its predicted positive association with ecosystem stability. In addition to having well identified species and strains and even clones, that allow to test reproductive effects (sexual versus asexual), *Artemia *benefits from the possibility to set up experimental testing at both laboratory scale and outdoor pond systems, for which a comprehensive cyst bank with sufficient amount of samples from all over the world is available.

## Background

Subscribers to the Convention on Biological Diversity agreed on a target of reducing the actual rate of biodiversity loss by 2010, at regional and national levels [1]. In looking for ways to assess progress towards this goal, discussion on how biodiversity should best be measured has intensified [2]. Biodiversity stands for biological variation at different hierarchical levels, i.e. from genetic diversity within species to diversity of ecosystems. But policy-makers tend to focus only on species richness which, whilst critically important, leaves aside the relevant process of population genetic divergence, accounting for the origin of biodiversity. Each population has the potential to become a new species depending on the circumstances experienced, say differential reproductive and dispersal success, population size fluctuations, natural selection and/or genetic drift [3]. Hence ability to monitor and maintain genetic population structure in a particular ecosystem over time requires adequate management of genetic diversity, to allow species persistence over time.

The 2010 goal requires stakeholders (government, the private sector, NGOs, individuals, scientists) to understand how ecosystem components interact to produce the services needed for development, and how severe perturbations could eventually jeopardize the expected benefits in the long run. This paper highlights the potential role inland saline lakes could play as relatively simple models to understand ecosystem dynamics in a way which can readily be understood by stakeholders, particularly policy-makers. At the same time, it emphasizes the role of the primitive salt-tolerant crustacean they contain, the brine shrimp *Artemia*, as a unique indicator species in monitoring biodiversity at the population level.

## Discussion

### Inland hypersaline lakes and *Artemia* as models

**Figure 1:**
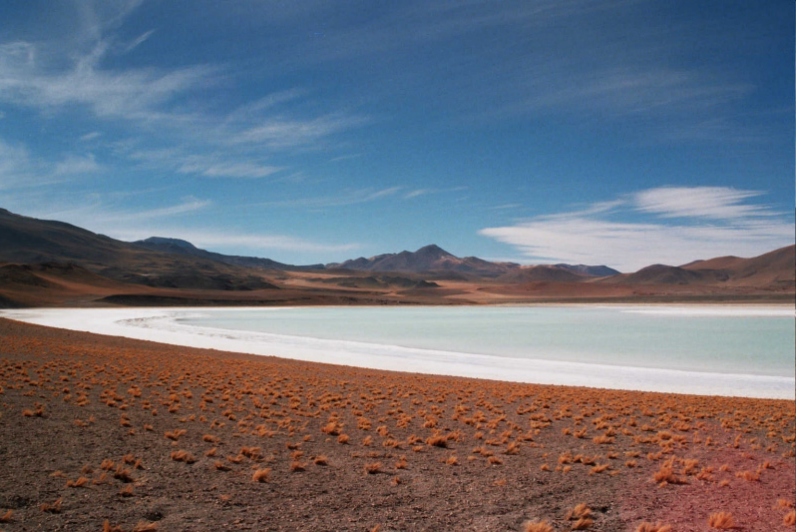
Salt lake in the Atacama Desert in northern Chile: a suitable biodiversity laboratory.

Inland hypersaline lakes found in arid and semi arid basins worldwide fulfil the condition of being relatively simple and manageable ecosystems which can be used to understand how their components interact, owing to reduced biodiversity and less complex structure as compared to almost all other systems including those in freshwater. Permanent salt lakes show an almost continuos range of salinity, from brackish to hypersaline, and all but those at the highest salinities are said to share some common features, and perhaps stressors (oxygen and temperature), with their freshwater counterparts [4]. Special cases are the athalassohaline lakes found in isolated evaporitic basins known as *salares *(saltflats) (see Figure 1) in the Atacama Desert of northern Chile (between 18° and 27° latitude South), reputedly one of the driest regions in the world (< 13 mm. annual rainfall). The area, a 700 kilometers-wide belt flanked on the one side by the Pacific Ocean and on the other by the Andes Mountains, is a unique setting since the saltflats and the water characteristics of lakes also vary depending on the associated salt deposits. Hence an almost continuous range of conditions is observed between the coastal range (Pacific Ocean) and the Altiplano salt deposits found up in the Andes, with the so-called Central and Pre-Andean depressions in between [5,6]. Key abiotic components are known (salinity, temperature, oxygen content and ionic composition) and are all amenable to monitoring on a regular basis [6]. Climatic and hydrological inputs (precipitation, surface runoff, groundwater inflow) and outputs (evaporation, drain losses) determine the salt content of the brines. Possession of reliable data on the hydrological regime and abiotic components could be of great help in predicting ecosystem response to stressors. We anticipate that *in situ *experiments using lake water in controlled containers should be possible. Multispecies trials could be run and possible stressors tested would be temperature, oxygen content, oscillations in salinity, restricted algal range, presence of non-*Artemia *competitor, Ca, Mg, K, NH4 ions. Such outdoor experiments in combination with laboratory ones can shed light on the effect of stressors, and If these are common to freshwater, i.e. oxygen and temperature, there are good chances to establish a link between both ecosystems.

Biodiversity composition of these systems is relatively simple as compared to middle and low salinity lakes. Some halo-tolerant bacteria and microalgal species are the diet of the brine shrimp *Artemia*, the dominant and most conspicuous inhabitant, whose abundance indicates water conditions and food availability. Within the limited range of ecological conditions of hypersaline lakes, *Artemia *is extremely successful achieving high population sizes and tolerating large environmental variation. Not surprisingly members of the genus are referred as the extremophiles of the biological world. For example the encysted gastrula embryo (cyst) is the most resistant of all animal life history stages, while the motile stages (nauplii and adults) are among the best osmoregulators in the animal kingdom [7]. Females switch reproductive mode (ovoviviparity vs. oviparity) as an adaptive response to environmental pressure, and hence this trait reflects water condition. Water characteristics and life history traits can be tested in ways that would be difficult to achieve in nature, if not impossible. Life history traits are easily dissected in individual females and tested in single or multiple environments under laboratory conditions [8]. The availability of an extremely comprehensive cyst bank with samples from all over the world (Artemia Reference Center in Ghent, Belgium) allows comparisons in common garden experiments. Additionally, *Artemia *offers a unique opportunity to set up experimental testing in outdoor pond systems, starting with well-identified species and even clones.

Because of these and other features *Artemia *is the organism of choice for different disciplines (evolutionary genetics, toxicology, radiation biology) and can be regarded as a sort of aquatic *Drosophila *[9].

### Biodiversity at population level

Organisms living in hypersaline lakes are expected to evolve relatively fast due to the mutagenic effect of the environment, for example, high UV radiation and salt concentration [10], nevertheless species richness of the genus is low. Only seven bisexual *Artemia *species (but many asexual types) have diverged so far from an ancestral form living in the Mediterranean area some 5 MYA, five in Eurasia (*A. salina*, *A. urmiana*, *A. tibetiana*, *A. sinica*, *A. spp*) and two in the Americas (*A. franciscana*, *A. persimilis*) [11,12]. In contrast, each species exhibits a highly heterogeneous population genetic structure, a pattern generated and maintained by environmental heterogeneity and the patchy (island-like) distribution of saline lakes. The homogenising impact of gene flux mediated by cyst dispersal by waterbirds or wind remains to be tested, as viability of cysts transferred into other lakes seems to be low [13]. Therefore, populations retain local adaptations that contribute to the long-term spatial and temporal persistence of genetic variants, some of which are associated with important life history traits. Monitoring population structure of a keystone species such as *A. franciscana*, the best studied so far, offers a simpler approach to evaluating the relationship between biodiversity and ecosystem stability than more complex, studies based on species richness. Biodiversity studies have been also performed with rotifer (*Brachionus*), in which sibling species may be frequent [14,15]. Unquestionably rotifers are a good model as well, but very few strains are available in the form of resting eggs, whilst the possibility to compare sexual and asexual types, an advantage of *Artemia*, is not possible in *Brachionus*. Furthermore there is considerably more information on genetic characteristics of *Artemia *than is available for rotifers.

A recent global concerted effort led by the Artemia Reference Center in Ghent (EU-supported project on Artemia Biodiversity) [16] resulted in a common conceptual background, intercalibrated molecular tools and methods to assess population genetic organization in different *Artemia *species around the world. The existing molecular database [12,17] covers a wide range of local populations of all bisexual species and asexual types, and therefore represents a good inventory of current *Artemia *biodiversity for future spatial and temporal monitoring of gene pools exposed to pollutants, transplantations and intensive and continued biomass harvest.

### Threats to *Artemia *biodiversity

Like other ecosystems, salt lakes and *Artemia *biodiversity face threats owing to their economic and natural environmental value. For example, global weather change affects rainfall and the hydrological cycle of salt and freshwater lakes, hence ecological and genetical models developed in the former could be useful in predicting response to these stressors in the latter. Mining activities often cause water diversion from salt lakes, a problem also affecting freshwater systems through agriculture and other economic activities (hydroelectric generation). Conflict of interest between stakeholders has become evident after some transnational mining companies are said to affect, as environmentalists and other interested parties believe, internationally important wetlands. Such is the case of Huasco saltflat in northern Chile, which is listed in Ramsar [18]. Non-economic value of salt lakes stems from being the habitat for migratory and breeding populations of waterbirds, particularly flamingos, and from recreational uses. Likewise, salt lakes are part of the historical landscape of indigenous people that populated the area some 13,000 years ago [19].

On the other hand, *Artemia *is traded as feed for aquaculture and ornamental fish, which brings about a subset of problems likely to affect *Artemia *biodiversity: through inoculation, transplantation, biomass and cyst harvest. For example loss of genotypes or co-adapted gene complexes occurs when an inoculated strain out-competes the local one. Some alleles present at different loci in the Macau (Brazil) population are absent in the probable founder population (San Francisco Bay). The Macau population exhibits new attributes such as tolerance to higher temperatures and cycles of cyst production as a consequence of the genetic reorganization in the introduced population. Gene pool modification is also possible through long-term artificial selective practices associated with exploitation (biomass harvest). Another source of risk is the widespread development of hatchery aquaculture, especially marine fish and shrimp, which demands significant amounts of *Artemia *as a diet for larval culture of many species [20]. Some hatcheries are located close to *Artemia *natural biotopes, hence these are likely to be affected by the escape via effluents of non-local *Artemia *strains (allochthonous). Finally, invasion by exotic species and gene pool depletion of different species by over-fishing are common in marine and fresh water environments. Using *Artemia *both processes can be studied in natural and laboratory conditions.

## Conclusion

Inland hypersaline lakes offer undisputable chances to assess progress in relation to the 2010 goal of reducing biodiversity loss. The Atacama Desert in northern Chile is highlighted as a special study area where athalassohaline lakes with unique ionic proportions abound. Their remoteness, low biodiversity, and identifiable abiotic components make them amenable to modelling and monitoring, and offer interdisciplinary research opportunities and management challenges. In fact, relatively simple systems are easier to dissect into their components and understand their interrelationships than more complex ones, like moderately saline or freshwater ecosystems. Besides, simple ecosystems should help policy-makers to understand how biodiversity is organized, maintained and how it should be managed on the long run. *Artemia *is considered a keystone species to evaluate biodiversity at the population level, i.e. spatial and temporal monitoring of genetic variants. Due to circumstances to which *Artemia *is exposed in nature, coupled to its advantages as a laboratory animal, it is an interesting model to study evolutionary response to extreme and heterogeneous environments. *Artemia *gene pools, in particular of *A. franciscana*, the best studied so far, are highly variable and are sub-structured in local populations differing in fitness-related and/or productive traits. Clearly, *Artemia *represents an example where conservation focus should be placed at the intraspecific level (local populations). *Artemia *habitats are extreme, old and hold a unique and simple biodiversity composition. As such they should be considered suitable natural biodiversity observatories (see Figure 1), in other words simple models to monitoring the dynamics of biodiversity at the intraspecific level. As salt lakes are said to share features and even stressors with other ecosystems, *Artemia *can be used to monitoring similar problems in other species. For example, colonizations or invasions of new habitats are examples of rapid evolutionary change amenable to testing in nature by exploiting the short generation time of *Artemia*, but can also be simulated readily under controlled laboratory conditions. Equivalent situations in other species are far more difficult to observe and study, either in nature or the laboratory. The impact of aquaculture species like salmonids that escape from enclosures and establish in non-native ecosystems would be an example [21].

There can thus, be little doubt that well planned projects could yield valuable information of both quantitative and qualitative nature on effects on biodiversity, of specific environmental stressors in the systems outlined above.

## Competing interests

The author(s) declare that they have no competing interests.

## Authors' contributions

GMG: is responsible for the design and first draft of the manuscript. PS and JAB substantially contributed to the idea of inland lakes as biodiversity observatories and *Artemia *as a model for intraspecific assesment of biodiversity, and contributed to its improvement by critically reading the manuscript. JAB also contributed to polishing the English style.
